# Hybrid kernels integrating genomic and multispectral data improve wheat genomic prediction accuracy

**DOI:** 10.1002/tpg2.70171

**Published:** 2025-12-30

**Authors:** Osval A. Montesinos‐López, Joel A. Martínez‐Regalado, Cintia Leonora Murillo‐Avalos, Andrew W. Herr, Abelardo Montesinos‐López, Iván Delgado‐Enciso, Jaime Cuevas, José Crossa, Arron H. Carter

**Affiliations:** ^1^ Facultad de Telemática Universidad de Colima Colima México; ^2^ Departamento de Estadística, Campus Miguel de Unamuno Universidad de Salamanca Salamanca Spain; ^3^ Centro Universitario de Análisis Estadísticos y de Opinión Pública (CUOP) Universidad de Colima Colima México; ^4^ Department of Crop and Soil Sciences Washington State University Pullman Washington USA; ^5^ Centro Universitario de Ciencias Exactas e Ingenierías (CUCEI) Universidad de Guadalajara Guadalajara Jalisco México; ^6^ School of Medicine University of Colima Colima Mexico; ^7^ División de Ciencias, Ingeniería y Tecnologías (DCIT) Universidad Autónoma del Estado de Quintana Roo Chetumal México; ^8^ Department of Statistics Post‐Graduate College (COLPOS) Montecillos Edo. México México

## Abstract

Genomic selection (GS) is transforming plant breeding by enabling more accurate and efficient identification of superior genotypes. However, its practical implementation remains challenging, as achieving high prediction accuracy is critical for its success. Several factors—including sample size, the degree of relatedness among individuals, and the complexity of target traits—significantly affect the predictive performance of GS models. To address these limitations, recent studies have explored the integration of genomic and phenomic information to enhance prediction accuracy. This integrated approach has shown promising results and continues to gain empirical support. In this study, we propose an alternative strategy to improve the efficiency and accuracy of GS by constructing hybrid kernels that combine genomic and phenomic information. Specifically, we generate two new kernels by combining the original genomic and phenomic kernels, aiming to capture complementary and previously unexploited sources of variation. We applied this approach to multi‐year data from the winter wheat (*Triticum aestivum* L.) breeding program at Washington State University, using phenomic data collected via unmanned aerial vehicles (UAVs). Our results provide empirical evidence that integrating genomic and UAV‐derived phenomic data through hybrid kernel modeling enhances the prediction accuracy of GS models. This approach achieved average improvements of 17.52%, 30.36%, 28.94%, and 16.73% in terms of Pearson's correlation, normalized root mean square error, and the percentage of correctly identified lines within the top 10% and 20%, respectively, compared with the conventional integration of genomic and phenomic information (M4 and M5). These findings highlight the potential of this method as a valuable and scalable tool for modern plant breeding programs.

## Core Ideas

- Integration of genomic and phenomic information to enhance prediction accuracy.
- Hybrid kernels, created from genomic and phenomic information—specifically, multispectral reflectance data collected via unmanned aerial vehicles (UAVs)—could be an approach that increases predictive performance of genomic selection models across years and environments.
- Phenomic information captures dynamic, environment‐influenced traits, providing a real‐time physiological snapshot of crop performance. By applying the hybrid kernel methodology to genomic and UAV‐derived phenomic data, we aim to evaluate whether this approach can further improve the predictive performance of genomic selection models across multiple years and environments in winter wheat (*Triticum aestivum* L.).

## Plain Language Summary

Practical implementation of genomic selection remains challenging, as achieving high prediction accuracy is critical for its success. Recent studies have explored the integration of genomic and phenomic information to enhance prediction accuracy. In this study, we constructed hybrid kernels that combine genomic and phenomic information for wheat. It was found that genomic prediction accuracy increases when using hybrid kernels.

**Abbreviations**
BLUE: best linear unbiased estimate
COR: average Pearson's correlation
NRMSE: normalized root mean square error
SNP: single nucleotide polymorphism
UAV: unmanned aerial vehicle

## 1 INTRODUCTION

Genomic selection (GS) has established itself as a fundamental tool in genetic improvement programs due to its ability to accelerate genetic progress and optimize efficiency in the selection of high‐yield cultivars and lines (Heffner et al., 2009). In a context where global population growth increases the demand for food, improving agricultural productivity and ensuring the sustainability of production systems has become a priority challenge. The development of genomic prediction (GP), proposed by Meuwissen et al. (2001), has allowed the optimization of the selection process in plant breeding, which traditionally consists of three fundamental phases: (i) the generation of genetic variability through directed crossings, (ii) the evaluation of plant material in various environments and crop cycles, and (iii) the selection of individuals with the greatest agronomic potential. The implementation of GP allows the prediction of the genetic merit of individuals in the early stages of the improvement process, significantly reducing the evaluation time and accelerating genetic gain per unit of time. This approach is particularly valuable in strategic crops such as wheat (*Triticum aestivum* L.), whose production is essential for global food security.

Statistical models used in GS have evolved significantly, with linear mixed models and their variants based on assumptions about the distribution of random effects being prominent. Among the most widely used methods are the ridge regression best linear unbiased predictor and the genomic best linear unbiased prediction (GBLUP), which use additive genomic relationship matrices to estimate breeding values (Endelman, 2011). However, despite advances in statistical modeling, the implementation of GS in breeding programs faces several limitations that affect its accuracy. Determining factors include the quality of genotyping, the size of the reference population, the correct distribution of individuals between the training and validation sets, the field design, the cross‐validation strategy, and the heritability of the trait analyzed (Crossa et al., 2017; Yoosefzadeh‐Najafabadi et al., 2022).

In addition, the agricultural sector faces unprecedented challenges due to climate change, including extreme temperatures, prolonged droughts, irregular rainfall, soil degradation, and scarcity of water resources (Atefi et al., 2021). To mitigate these impacts, genetic improvement programs seek to develop cultivars with greater stress tolerance, resistance to pests and diseases, and greater efficiency in the use of resources (Fischer, 2009; Rahaman et al., 2015). In this sense, the integration of high‐throughput phenotypic information with genomic data has been proposed as a key strategy to improve the accuracy of GS. Advanced technologies such as remote sensing, unmanned aerial systems (UAS), portable scanners, tractor‐mounted sensors, and low‐orbit satellites have allowed obtaining detailed and real‐time phenotypic information (O. A. Montesinos‐López et al., 2023).

Empirical evidence indicates that combining high‐throughput phenotypic data with genomic information can significantly optimize GS and improve agricultural productivity. Recent studies have shown that the integration of transcriptomics, genomics, and metabolomics increases predictive ability in diverse crops (Hu et al., 2021; P. Y. Wu et al., 2022). Furthermore, since the phenotypic variability observed in different environments is the result of the interaction between genetic and environmental factors, the inclusion of high‐resolution environmental data in prediction models is crucial to optimize the selection of cultivars adapted to diverse agroclimatic conditions (Rogers et al., 2021). The integration of historical and real‐time data allows for more accurate modeling of genotype‐environment interactions, which improves the efficiency of breeding programs and contributes to the development of a more resilient and sustainable agriculture (Messina et al., 2018; Millet et al., 2019).

To overcome these limitations, recent efforts have focused on developing more flexible kernel‐based approaches that go beyond traditional additive frameworks. Among these, a particularly promising advancement is the method proposed by Cuevas et al. (2025), which introduces a novel way to construct hybrid kernels by combining information through matrix products rather than simple summation. Recently, they introduced an innovative framework for enhancing GP by combining different sources of relationship information through hybrid kernels. Rather than using traditional additive kernel combinations (e.g., simple summation of genomic and pedigree matrices), their approach involved computing the upper and lower triangular products of two kernel matrices, thereby constructing two new hybrid kernels: one based on the upper triangle of the matrix product and one based on the lower triangle. This method allowed for the capture of nonredundant, complementary patterns of similarity that are not modeled when kernels are simply added together. They demonstrated that this approach improves prediction accuracy, particularly in settings with sparse testing and unbalanced data, where standard methods often struggle to fully exploit existing information.

The current study builds directly upon this kernel construction framework. While Cuevas et al. (2025) focused on combining genomic and pedigree kernels, here we extend the method to combine genomic and phenomic kernels derived from unmanned aerial vehicle (UAV)‐based multispectral imaging. In doing so, we not only test the generalizability of the hybrid kernel concept but also assess its performance when integrating two fundamentally different types of biological information—one reflecting inherited genetic potential and the other reflecting phenotypic expression shaped by environment. This extension is particularly valuable for plant breeding programs aiming to leverage high‐throughput phenotyping (HTP) tools for improved GP.

While individual traits differ in complexity and heritability, the integration of multispectral phenomics with genomics is a modeling innovation expected to benefit phenotypically diverse targets. Thus, reporting performance summarized across traits offers a concise and statistically stable indicator of hybrid kernel effectiveness. This prevents misleading trait‐by‐trait contrasts driven by local environmental noise and ensures that conclusions reflect the overall breeding value of the method.

Cuevas et al. (2025) demonstrated that such hybrid kernels, created from genomic and pedigree information, significantly enhance prediction accuracy, especially in unbalanced and sparse‐testing scenarios common in practical breeding programs. Building upon this foundation, the present study extends the hybrid kernel framework to integrate genomic and phenomic information—specifically, multispectral reflectance data collected via UAV. Unlike pedigree data, phenomic information captures dynamic, environment‐influenced traits, providing a real‐time physiological snapshot of crop performance. By applying the hybrid kernel methodology to genomic and UAV‐derived phenomic data, we aim to evaluate whether this approach can further improve the predictive performance of GS models across multiple years and environments in winter wheat. This integration not only tests the versatility of this framework but also addresses the growing need for scalable methods that combine HTP and genomic information in modern plant breeding. For this purpose, data collected between 2019 and 2022 by Washington State University (WSU) were used, which were analyzed under a comparative approach using the GBLUP and transfer methods to evaluate their predictive efficiency.

**Core Ideas**

- Integration of genomic and phenomic information to enhance prediction accuracy.
- Hybrid kernels, created from genomic and phenomic information—specifically, multispectral reflectance data collected via unmanned aerial vehicles (UAVs)—could be an approach that increases predictive performance of genomic selection models across years and environments.
- Phenomic information captures dynamic, environment‐influenced traits, providing a real‐time physiological snapshot of crop performance. By applying the hybrid kernel methodology to genomic and UAV‐derived phenomic data, we aim to evaluate whether this approach can further improve the predictive performance of genomic selection models across multiple years and environments in winter wheat (*Triticum aestivum* L.).

## 2 MATERIALS AND METHODS

### 2.1 Data

#### 2.1.1 Phenotypic data

Datasets 1 through 3, collectively referred to as the *wheat datasets*, were employed in this study. These datasets are the same as those used in the article by O. A. Montesinos‐López et al. (2023), titled “*Genomics combined with UAS data enhances prediction of grain yield in winter wheat*.” They were analyzed for the grain yield (GY) trait, plant height (PH), and days to heading. The wheat lines included in these datasets were developed by the WSU breeding program and were cultivated at various sites across the state of Washington. A brief description of each dataset is provided below:

1. Dataset 1: Wheat_1 (2019): This dataset comprises 1379 unique wheat lines, each evaluated in one of two environments—Kincaid or Pullman. The total number of observations is 1379, with each line assessed in only a single environment, meaning no replication across environments.
2. Dataset 2: Wheat_2 (2020): This dataset contains 758 distinct wheat lines evaluated across six environments: Farmington, Harrington, Kincaid, Lind, Ritzville, and Walla Walla. Due to repeated testing of some lines in multiple environments, the dataset includes a total of 952 observations.
3. Dataset 3: Wheat_3 (2021): Comprising 452 unique wheat lines, this dataset covers eight environments: Davenport, Harrington, Kahlotus, Kincaid, Lind, Pullman, Ritzville, and Walla Walla. A total of 780 observations are included, as some lines were tested in more than one environment.

For each dataset, best linear unbiased estimates (BLUEs) were computed using either an alpha lattice design or an augmented randomized complete block design. These experimental designs enabled the estimation of unbiased trait means for downstream modeling. For further details on the computation of BLUEs, refer to O. A. Montesinos‐López et al. (2023). The data are available at: https://github.com/osval78/Integrating_Genomic_and_Phenomics.

#### 2.1.2 Multispectral data

To acquire multispectral data, the Sentera Quad Multispectral Sensor (Sentera) was utilized. This device integrates four individual sensors capturing eight wide spectral bands ranging from 450 to 970 nm—wavelengths previously identified as relevant for evaluating winter wheat in Washington State (Sankaran et al., 2015). The sensor was mounted on a DJI Inspire 1 UAV, which flew along a predefined flight path at an altitude of 45 m. During each flight, georeferenced images were captured with approximately 85% image overlap. Data acquisition was performed within a 4‐h window centered on solar noon, aiming to minimize variation in solar radiation by flying as close to solar noon as possible. Flights typically lasted about 20 min and were conducted on days with clear skies to ensure consistent lighting conditions. Imaging was conducted during the Feekes growth stages 10.1–10.5 (more details in O. A. Montesinos‐López et al., 2024).

The images collected by the UAV were processed using Pix4Dmapper software (Pix4D Inc., 2023) to generate an orthomosaic for each sensor at each location. These orthomosaics were then imported into the QGIS Geographic Information System for plot delineation and were further processed with a custom R script. This processing included radiometric calibration, calculation of spectral indices, and extraction of average values for each plot.

In the 2019 season, radiometric calibration was performed using a single reflectance panel with 85% reflectance for the RGB (red, green, and blue spectral bands) and red edge bands (Sankaran et al., 2015). For the near‐infrared (NIR) band, normalization was conducted using a correction factor of 3.07 based on the formula:

$$\mathrm{NIR}=\left(2.921\times \;\mathrm{Blue}\right)-\left(0.754\times \mathrm{Red}\right).$$

Between 2020 and 2022, a set of five reflectance calibration panels (ranging from 2% to 85% reflectance; MosaicMill Oy) was used. Raw digital number (DN) values from each band were calibrated using a linear transformation:

SR=DN×Slope±Intercept,

where SR denotes the surface reflectance, and the slope and intercept were obtained from regression models fitted to the known reflectance values of the calibration panels. These adjusted reflectance values were then used for index calculations in downstream model analyses. The computed indices were Can_Cover, normalized difference red edge 1, and normalized difference vegetation index. Equations used in the calculation of indices can be found in Table 1. For more details of the multispectral bands used, see O. A. Montesinos‐López et al. (2023) and O. A. Montesinos‐López et al. (2024). We consistently use the notation kernel multispectral (K_H) instead of the perhaps more appropriate kernel phenomic (K_P) in tables and results to emphasize its predictive role rather than strict spectral granularity.

**TABLE 1:** Spectral reflectance indices implemented.

Spectral reflectance indices	Abbreviation	Equation	Reference
Normalized difference vegetation index	NDVI	$\frac{R800-R680}{R800+R680}$	Rouse et al. (1973)
Normalized difference red edge 1	NDRE1	$\frac{R800-R700}{R800+R700}$	Gitelson and Merzlyak (1996)
Percent canopy coverage	Can_Cover	$\frac{1}{N}\sum_{i=1}^{N}\mathrm{GNDV}I_{i}$	Sankaran et al. (2015)

Note: The first column provide the name of the spectral indices, the second one its abbreviation, the third one the equation used for computing each indice and the last one the reference for each indices.

#### 2.1.3 Genomic data

For genotypic characterization, all wheat lines were subjected to genotyping‐by‐sequencing (Poland et al., 2012). The initial dataset contained 6,075,743 single nucleotide polymorphisms (SNPs). This set was filtered to retain only informative markers, excluding SNPs with more than 80% homozygosity, more than 50% missing data, a minor allele frequency below 0.05, or heterozygosity exceeding 5%. After filtering, 19,645 SNPs remained for analysis. To handle missing marker data, imputation was performed using the expectation‐maximization algorithm implemented in the R package rrBLUP (Endelman, 2011). This procedure ensured a complete dataset for subsequent analyses.

### 2.2 Statistical models

Models M1–M8 were implemented and described in the next sections. Also, a full description of the models is given in Table 2.

**TABLE 2:** Genomic prediction models and their kernel configurations.

Model	Original name	Simplified name	Kernel(s) used	Description
M1	G	Linear‐Genomic (LG)	K_G	Linear genomic kernel (e.g., VanRaden, 2008) from SNP data.
M2	H	Linear‐Phenomics (LP)	K_H	Linear multispectral kernel from UAV reflectance data.
M3	K	Gaussian‐Genomic (GG)	K_GG	Gaussian kernel applied to genomic data.
M4	KH	Linear‐Geno‐Phenomic (LGP)	K_GG + K_H	Additive model combining linear genomic and phenomic kernels.
M5	KK_H	Mixed‐Gaussian (MGL)	K_GG + K_HG	Additive model using Gaussian genomic and Gaussian phenomic kernels.
M6	KHCP	Hybrid‐Product (HP1)	K_H + K_GG + K_UT + K_LT	Hybrid model. K_UT and K_LT are derived from matrix product K_H × K_GG.
M7	KHCPH	Hybrid‐Gaussian (HP2)	K_H + K_GG + K_UT + K_LT	Same as HP1 but using Gaussian multispectral kernel K_HG.
M8	KK_H_CPH	Hybrid‐Gauss+ (HP3)	K_HG + K_GG + K_UT + K_LT	Full hybrid with Gaussian kernels and hybrid triangular terms K_UT and K_LT are obtained from the matrix product K_HG × K_GG.

*Note*: K_G: Linear genomic kernel (e.g., VanRaden matrix); K_H: Linear kernel from multispectral reflectance data; K_GG: Gaussian kernel from genomic data; K_HG: Gaussian kernel from multispectral data; K_UT and K_LT: Upper and lower triangular matrices derived from the matrix product of K_H × K_GG (in HP1), or K_HG × K_GG (in HP2 and HP3). These triangular kernels capture asymmetric interactions between genomic and phenomic information; Only models M6, M7, and M8, HP1, HP2, and HP3 involve matrix products for hybrid kernel construction, based on the methodology described by Cuevas et al. (2025).

### 2.3 Models 1 to 3 (M1–M3)

The predictive response model was considered to be:

(1) $$Y_{ij}=\mu +g_{j}+\varepsilon_{ij},$$

where $Y_{ij}$ denotes the response variable measured at genotype j at replication i, i=1,…,I; μ denotes a general mean; $g_{j},j=1,\text{…},J$, are the random effects of lines and *ϵ*_ij_ denotes the random error terms assumed normally distributed with mean 0 and variance $\sigma^{2}$. Furthermore, it is assumed that $g={\left(g_{1},\text{…},g_{J}\right)}^{T}\;∼N_{J}\left(0,\sigma_{g}^{2}G\right)$, G denotes the linear kernel computed as $G=\frac{XX^{T}}{p}$ or order J×J this matrix is called the genomic relationship matrix (VanRaden, 2008) in plant and animal breeding since it is computed with a standardized matrix of SNPs (X) coded as 0, 1, and 2 of order or order J×p, where p denotes the number of markers. For this reason, the G matrix is assumed to be known before starting the modeling process. Then the model given in Equation (1) was implemented with the linear kernel, G, this model is denoted as model **G** (model 1, M1). But when the model given in Equation (1) was implemented with a linear kernel or order J×J using the multispectral information $H=UU^{T}/3$, where U denotes the matrix of standardized multispectral information of order J×3, this model was called **H** (M2). On the other hand, when with the marker information was computed a Gaussian kernel K of order J×J the model is denoted as **K** (M3). The Gaussian kernels were computed as $k\left(x_{j},x_{k}\right)=\exp\left(-\frac{{∥x_{j}-x_{k}∥}^{2}}{\sigma^{2}}\right)$, where $x_{j}$ and $x_{k}$ are two p dimensional vectors coming from the standardized matrix of SNPs (X), p represent the number of SNPs, while i and j represent two genotypes (individuals), and $\sigma^{2}$ is the bandwidth of the kernel that was computed as the median of all pairwise squared distances between individuals. When the data come from multispectral information, we replace the marker data with this information.

### 2.4 M4 and M5

The model KH (denoted as M4) combines both of the predictor kernels that define models M2 and M3, that is, the same kernels employed in those models,

(2) $$Y_{ij}=\mu +g_{Hj}+g_{Gj}+\varepsilon_{ij,}$$

where $Y_{ij}$ and $\varepsilon_{ij}$ were described as in Models 1 to 3. Also $g_{Hj}$ is described as in M2, that is, $g_{H}={\left(g_{1},\text{…},g_{J}\right)}^{T}\;∼N_{J}\left(0,\sigma_{g}^{2}H\right)$. In a similar fashion $g_{Gj},j=1,\text{…},J$, denotes random effects of lines but under a Gaussian kernel, that is, $g_{G}={\left(g_{1},\text{…},g_{J}\right)}^{T}\;∼N_{J}\left(0,\sigma_{g}^{2}K\right)$, where K denotes the Gaussian kernel described in model M3. Model M5 (denoted as KK_H) is equal to model M4 except that the $g_{H}$ term that used the multispectral information with the linear kernel H, used a Gaussian kernel, $K_{H}$ or order J×J computed with the multispectral information.

### 2.5 Model M6 to M8

Model M6 denoted as KHCP, is equal to M4 but with two additional terms that result from combining the multispectral linear and marker Gaussian kernel. For this reason, the model is equal to:

(3) $$Y_{ij}=\mu +g_{Hj}+g_{Gj}+g_{CCj}+g_{PPj}+\varepsilon_{ij},$$

where $Y_{ij}$, $g_{Hj},g_{Gj}$ and $\varepsilon_{ij}$ were described in M4, while $g_{CCj},j=1,\text{…},J$ are random effects of lines distributed as $g_{CC}={\left(g_{1},\text{…},g_{J}\right)}^{T}∼N_{J}\left(0,\sigma_{gCC}^{2}K^{CC}\right)$ and $g_{PPj},j=1,\text{…},J$ are random effects of lines distributed as $g_{PP}={\left(g_{1},\text{…},g_{J}\right)}^{T}∼N_{J}\left(0,\sigma_{gPP}^{2}K^{PP}\right)$. Where $\sigma_{gCC}^{2}$ is the variance component of lines associated with the random effect $g_{CC}$, and $\sigma_{gPP}^{2}$ is the variance component of lines associated with the random effect $g_{PP}$. While $K^{\mathrm{CC}}$ and $K^{\mathrm{PP}}$ both of order or order J×J are subproducts of the matrix multiplication of the multispectral linear (H) and marker Gaussian (K) kernels, for this reason the $K^{\mathrm{CC}}$ is computed using the upper triangular part (**UT**) of the matrix multiplication of H and K, that is, $K^{\mathrm{CC}}=$ **UT** + t(**UT**). While the kernel $K^{\mathrm{PP}}$ was computed using the lower part (**LT**) of the matrix multiplication of H and K, that is, $K^{\mathrm{PP}}=$ **LT**+t(**LT)** (Cuevas et al., 2025). Model M7 (denoted as KHCPH) is similar to model M6 except that the computation of the last two terms involving the $K^{\mathrm{CC}}$ and $K^{\mathrm{PP}}$ kernels were computed from the subproducts of the matrix multiplication between the multispectral ($K_{H})$ and marker (K) Gaussian kernels and the new kernels corresponding to $K^{\mathrm{CC}}$ and $K^{\mathrm{PP}}$ were denoted as $K^{\mathrm{CCH}}$ and $K^{\mathrm{PPH}}$ and also both are of order J×J. Finally, model M8 (denoted as KK_H_CPH) is equal to model M7, except that its first term that contains the linear multispectral kernel was replaced for its corresponding Gaussian multispectral kernel, $K_{H}$. All these models had been implemented in the R statistical software (R Core Team, 2025) utilizing the BGLR library (Pérez & de los Campos, 2014).

All models included in this study are described and summarized in Table 1. It is important to note that these models were implemented under two types of analysis: (a) uni‐environment and (b) multi‐environments setting. In the uni‐environment case, the eight models described above were applied one environment at a time; in other words, the implementation used data from only a single environment (with the subscript *i* taking the value of just one environment). In contrast, the multi‐environments implementation used all environments simultaneously, that is, *i* = 1, …, *I*. However, it should be emphasized that in the multi‐environments setting, the modeling process treats the environments as replications. This indicates that the BLUEs were calculated separately for each environment, as pointed out in O. A. Montesinos‐López et al. (2023).

Finally, it is worth noting that the models considered here include both linear kernels (GBLUP) and Gaussian kernels. The linear kernel assumes additive relationships and provides a computationally efficient baseline, whereas the Gaussian kernel can capture potential nonlinear genotype–phenotype interactions by implicitly mapping data into higher dimensional feature spaces. Including both kernel types enables a broader evaluation of scenarios where linear effects suffice and cases where nonlinear modeling may offer additional benefits.

### 2.6 Cross‐validation and evaluation metrics

To evaluate and compare the predictive performance of the models, a cross‐validation (CV) approach was employed. The strategy simulated a CV1 scenario of untested lines in tested environments. It was implemented using five random partitions of the full dataset, with each partition allocating 80% of the observations for training and the remaining 20% for testing (Alemu et al., 2024; O. A. Montesinos‐López et al., 2022). Predictive performance was assessed using four metrics: normalized root mean square error (NRMSE) where the NRMSE is a standardized version of the root mean square error, which allows you to compare prediction errors across datasets with different scales or across traits for the same dataset with different scales. It measures how far predicted values deviate from observed values, relative to the range or mean of the observed data. In this case, its computation was performed relative to the mean of observed data. Although MAPE is a scale‐independent error metric widely used for comparing performance across traits, we focused on NRMSE here for consistency with previous GP studies. Average Pearson's correlation (COR) and the proportion of correctly identified top‐performing lines within the top 10% (PM_10) and top 20% (PM_20) of the testing sets. For each model, the average values of NRMSE, COR, PM_10, and PM_20 were computed across all partitions, and reported as the overall prediction accuracy for each dataset. For comparison of the models in each year and across years, we computed the relative efficiency (RE) in percentage for each metric. For COR was computed as follows:

(4) $$RE_{\mathrm{COR}}=\left(\frac{\mathrm{CO}R_{\mathrm{bm}}}{\mathrm{CO}R_{k}}\;-\;1\right)\times 100,$$

where $\mathrm{CO}R_{\mathrm{bm}}$ denotes the COR of the best model and $COR_{k}$ denotes the COR of any other of the remaining models. For the NRMSE was computed with:

(5) $$RE_{\mathrm{NRMSE}}=\left(\frac{\mathrm{NRMSE}R_{k}}{\mathrm{NRMSE}R_{\mathrm{bm}}}-\;1\right)\times 100,$$

whereNRMSE denotes the $\mathrm{NRMSE}R_{\mathrm{bm}}$ of the best model and $\mathrm{NRMSE}R_{k}$ denotes the NRMSE of any other of the remaining models. For PM_10_, we used the following expression:

(6) $$RE_{PM_{10}}=\left(\frac{PM_{10\mathrm{bm}}}{PM_{10k}}\;-\;1\right)\times 100,$$

where ${\mathrm{PM}}_{10}$ denotes the ${\mathrm{PM}}_{10}$ of the best model and ${\mathrm{PM}}_{10k}$ denotes the ${\mathrm{PM}}_{10}$ of any other of the remaining models. Finally, forMP_20_, we used the following expression:

(7) $$RE_{PM_{20}}=\left(\frac{P{M_{20}}_{\mathrm{bm}}}{P{M_{20}}_{k}}-\;1\right)\times 100,$$

where$\;{\mathrm{PM}}_{20\mathrm{bm}}$ denotes the $PM_{20}$ of the best model and ${\mathrm{PM}}_{20k}$ denotes the $PM_{20}$ of any other of the remaining models.

It is important to note that, given the structure of the datasets—each comprising multiple environments—the statistical models described in the Section 2.2 were implemented in two distinct ways:

1. *Single‐environment approach*: Models were fitted independently for each environment within each dataset;
2. *Multi‐environment approach*: Models were applied to the entire dataset, treating environments as replications.

Only the models outlined in Section 2.2 were implemented due to the highly unbalanced nature of the datasets, where the number of shared lines across environments within each dataset is very limited.

## 3 RESULTS

**FIGURE 1:**
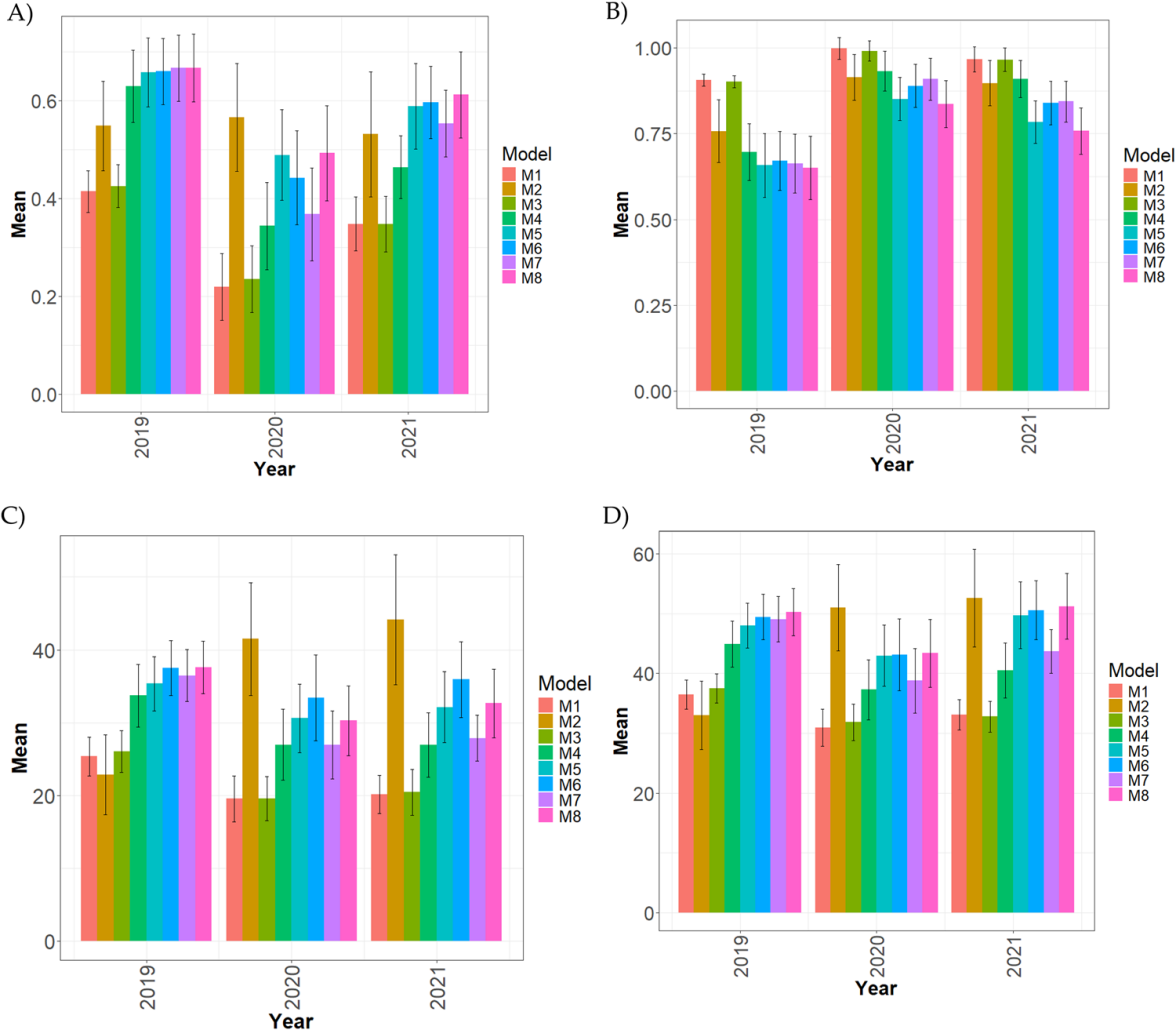
Comparative performance of genomic prediction models in a single‐environment framework for the Wheat_WSU dataset, summarized across environments and traits. Results are shown for four evaluation metrics: (A) Pearson's correlation (COR), (B) normalized root mean square error (NRMSE), (C) percentage of matching in the top 10% (PM_10), and (D) percentage of matching in the top 20% (PM_20).Higher values of COR, PM_10_, and PM_20_, and lower values of NRMSE, indicate better model performance. The performance of each model (M1–M8) is compared across these metrics to identify the most effective approach for genomic prediction under single‐environment conditions.

**FIGURE 2:**
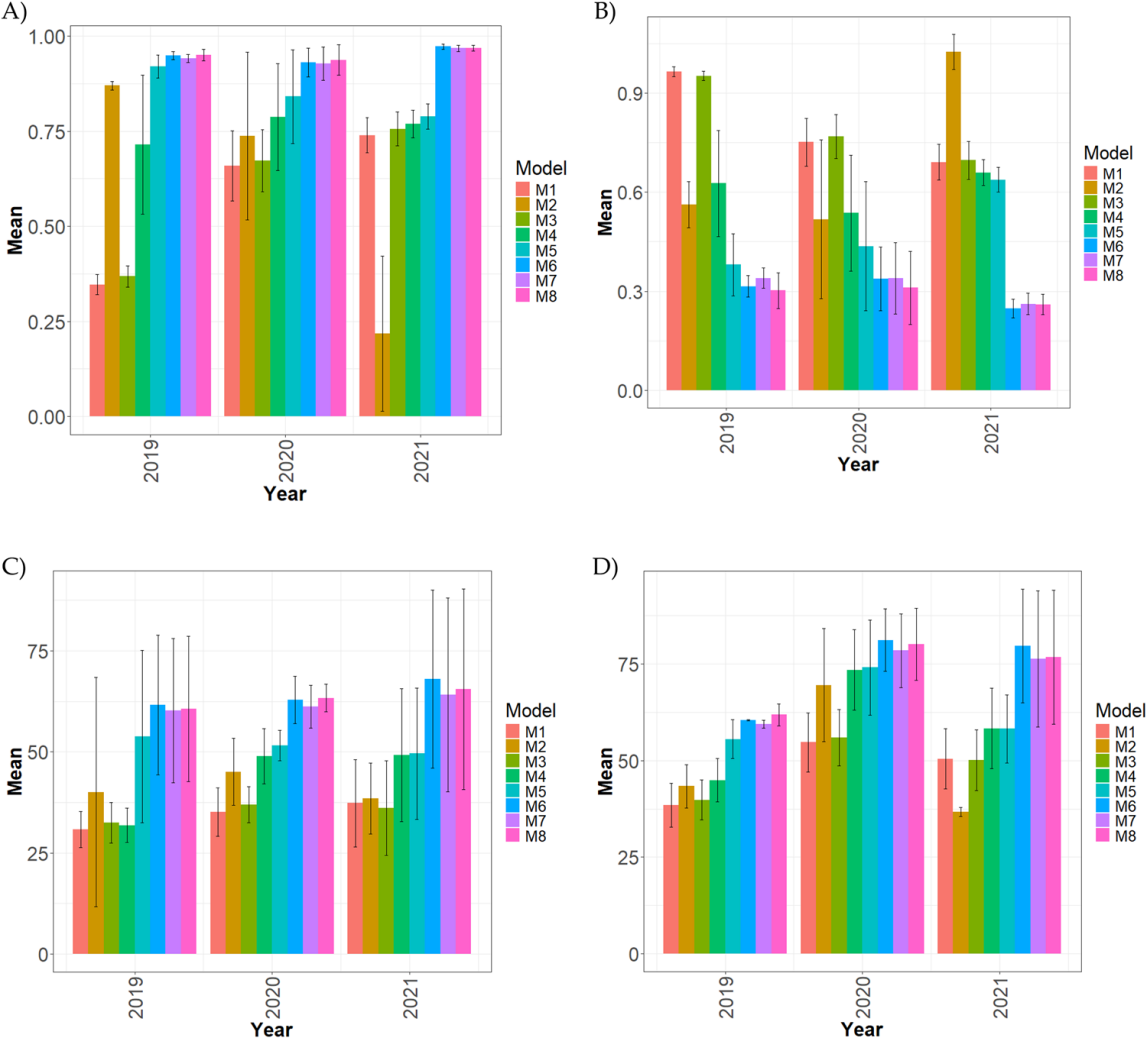
Comparative performance of genomic prediction models in a multi‐environment framework for the Wheat_WSU dataset, summarized across environments and traits. The evaluation metrics include: (A) Pearson's correlation (COR), (B) normalized root mean square error (NRMSE), (C) percentage of matching in the top 10% (PM_10), and (D) percentage of matching in the top 20% (PM_20). Results highlight the models’ predictive performance under genotype × environment interactions. Higher COR and PM values, and lower NRMSE, indicate better generalization across environments. Model M8 consistently demonstrated superior performance across most metrics.

**FIGURE A1:**
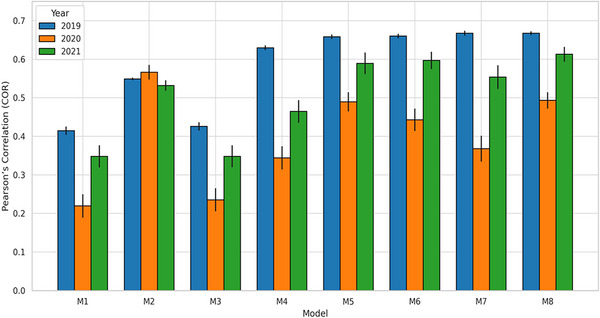
Bar plot showing the average Pearson's correlation (COR) for genomic prediction models M1 through M8 under the uni‐environment framework across the years 2019, 2020, and 2021. Each bar represents the mean COR value for a specific model in a given year, and error bars indicate the standard error (SE) of the correlation estimate. Models integrating both genomic and multispectral information (e.g., M6, M7, and M8) tend to show consistently higher correlations with lower SEs, especially in later years, compared to models using only genomic (M1 and M3) or only multispectral data (M2).

**FIGURE A2:**
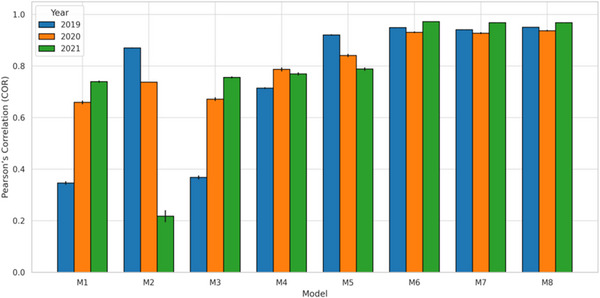
Comparative performance of eight genomic prediction models (M1–M8) across 3 years (2019, 2020, and 2021) in a multi‐environment prediction framework. The bar plot shows the average Pearson's correlation (COR) coefficient between observed and predicted values for each model and year. Error bars represent the standard error (SE) of the correlation estimates. Models M1–M3 are based on genomic (*G*), multispectral (*H*), or kernel (*K*) inputs; M4 and M5 incorporate intermediate combinations of data sources; while M6–M8 integrate multiple sources including genomic, multispectral, canopy, and physiological data. Overall, models M6 to M8 show consistently higher COR values across years, reflecting improved predictive performance when combining multiple data types.

The results are presented in two sections. The first section provides the results for the three datasets implanted under a *Single‐environment* framework. The second section describes the results of the models evaluated under a *Multi‐environment* framework. In both analyses, the performance of the GP models is compared in terms of Pearson's correlation (COR), NRMSE, and percentage of matching in the top 10% (PM_10) and top 20% (PM_20). Tables A1 and A2 contain the results shown in Figures 1 and 2. Figure A1 show the results for each correlation in each year and single environment, and Figure A2 display the correlation results for each year but across environments. It is important to note that in the single‐ and multi‐environment analyses, results are reported across environments and traits.

### 3.1 Single‐environment results

In 2019 (Figure 1A), M7 and M8 achieved the highest correlation (0.667), and M6 (0.660) showed nearly identical performance. M5 (0.658) was also close, while M4 dropped more sharply (0.630, −5.87.0%). M2 showed a substantial reduction (0.549, −21.49%), and the weakest results were recorded for M3 (0.426, −56.57%) and M1 (0.415, −60.72%).

In 2020, substantial discrepancies appeared (Figure 1A). M2 emerged as the leading model (0.566). M8 (0.493, −14.81%) and M5 (0.489, −15.74%) followed at a distance, while M6 (0.443, −27.769%) and M7 (0.368, −53.80%) performed considerably worse. Extreme underperformance was observed in M3 (0.235, −140.85%) and M1 (0.220, −157.270%).

In 2021 (Figure 1A), M8 regained superiority (0.613), with M6 (0.597, −2.68%) and M5 (0.589, −4.07%) remaining competitive. M7 (0.554, −10.65%) and M2 (0.532, −15.23%) performed moderately, while M4 dropped more sharply (0.464, −32.11%). M3 (0.348, −76.15%) and M1 (0.348, −76.15%) again showed the weakest results.

For NRMSE (Figure 1B; Table A1), M8 achieved the lowest error in 2019 (0.651), 2020 (0.836), and 2021 (0.758). In 2020, the next best performers were M5 (0.851, +1.79%) and M6 (0.89, +6.46%), followed by M7 (0.909, +8.73%), M2 (0.915, +9.45%), and M4 (0.932, +11.48%). The highest errors were consistently observed for M3 and M1: 0.901 and 0.906 in 2019 (+38.40% and +39.17%), 0.991 and 0.998 in 2020 (+18.54% and +19.38%), and 0.966 and 0.967 in 2021 (+27.44% and +27.57%), all relative to the yearly best.

Regarding PM_10 (Figure 1C), M8 dominated in 2019 (37.593), whereas M2 clearly surpassed all others in 2020 (41.485) and 2021 (44.115). The largest performance gaps were observed in M1 and M3, which in all years recorded reductions exceeding 44% relative to the best models.

The PM_20 analysis (Figure 1D) confirmed these patterns: M8 led in 2019 (50.237), but M2 became the strongest in 2020 (51.015) and 2021 (52.600). In contrast, M1 and M3 consistently showed the weakest predictive capacity, with differences reaching −33% or worse.

Overall, three trends emerge: (1) M8 was the most stable and competitive across years, especially in 2019 and 2021; (2) M2 occasionally outperformed all others, particularly under PM‐based measures and in 2020; and (3) M1 and M3 systematically underperformed, with extreme percentage gaps relative to the best models.

### 3.2 Multi‐environmental results

Figure 2 presents the multi‐environmental results of the proposed models in terms of COR, NRMSE, and the percentage of matching at top 10% (PM_10) and top 20% (PM_20). For more details, see Table A2.

In 2019 (Figure 2A), M8 achieved the highest correlation (0.951), followed very closely by M6 (0.949, −0.16%) and M7 (0.941, −1.00%). M5 (0.921) showed a moderate decrease (−3.25%). In contrast, M2 (0.870, −9.24%) and M4 (0.715, −33.03%) performed substantially worse, whereas M3 (0.368, −158.36%) and M1 (0.346, −174.63%) displayed extreme underperformance.

In 2020 (Figure 2A), M8 again led (0.937), followed by M6 (0.931, −0.63%) and M7 (0.928, −1.00%). M5 (0.841, −11.43%) lagged further behind, whereas M4 (0.787, −19.07%) and M2 (0.737, −27.08%) showed poor results. The weakest models were M3 (0.672, −39.49%) and M1 (0.659, −42.20%).

In 2021 (Figure 2A), M6 became the leading model (0.972), slightly ahead of M8 (0.969, −0.37%) and M7 (0.968, −0.41%). M5 (0.789, −23.3%) and M4 (0.769, −26.37%) showed sharp decreases, while M3 (0.756, −28.63%) and M1 (0.739, −31.48%) performed poorly. M2 (0.218, −346.58%) exhibited extreme instability.

For NRMSE (Figure 2B), M8 had the lowest values in 2019 (0.302) and 2020 (0.311), whereas M6 performed best in 2021 (0.249). Differences among M6, M7, and M8 were minimal (≤13%). In contrast, M1 and M3 showed consistently high error levels (≥0.75 in 2020, ≥0.69 in 2021), while M2 presented extreme instability in 2021 (1.024, +311.95%).

PM_10 results (Figure 2C) showed M6 as the best performer in 2019 (61.622) and 2021 (68.063), while M8 led in 2020 (63.333). M6, M7, and M8 consistently ranked at the top, whereas M1, M2, M3, and M4 showed large decreases, often exceeding −29%, with the weakest models approaching −100%.

For PM_20 (Figure 2D), M8 led in 2019 (61.860), while M6 was the strongest in 2020 (78.456) and 2021 (79.635). M7 remained consistently close to the leaders. In contrast, M1, M2, and M3 repeatedly showed weak predictive capacity, with reductions ranging from −16% to more than −100% in some cases.

Overall, the multi‐environmental analysis reveals three key patterns: (1) M6 and M8 were consistently among the top‐performing models, often separated by minimal differences; (2) M2 was unstable, with particularly extreme underperformance in 2021; and (3) M1 and M3 systematically ranked as the weakest models across all metrics.

### 3.3 Across environments, traits, years, and type of analysis

**FIGURE 3:**
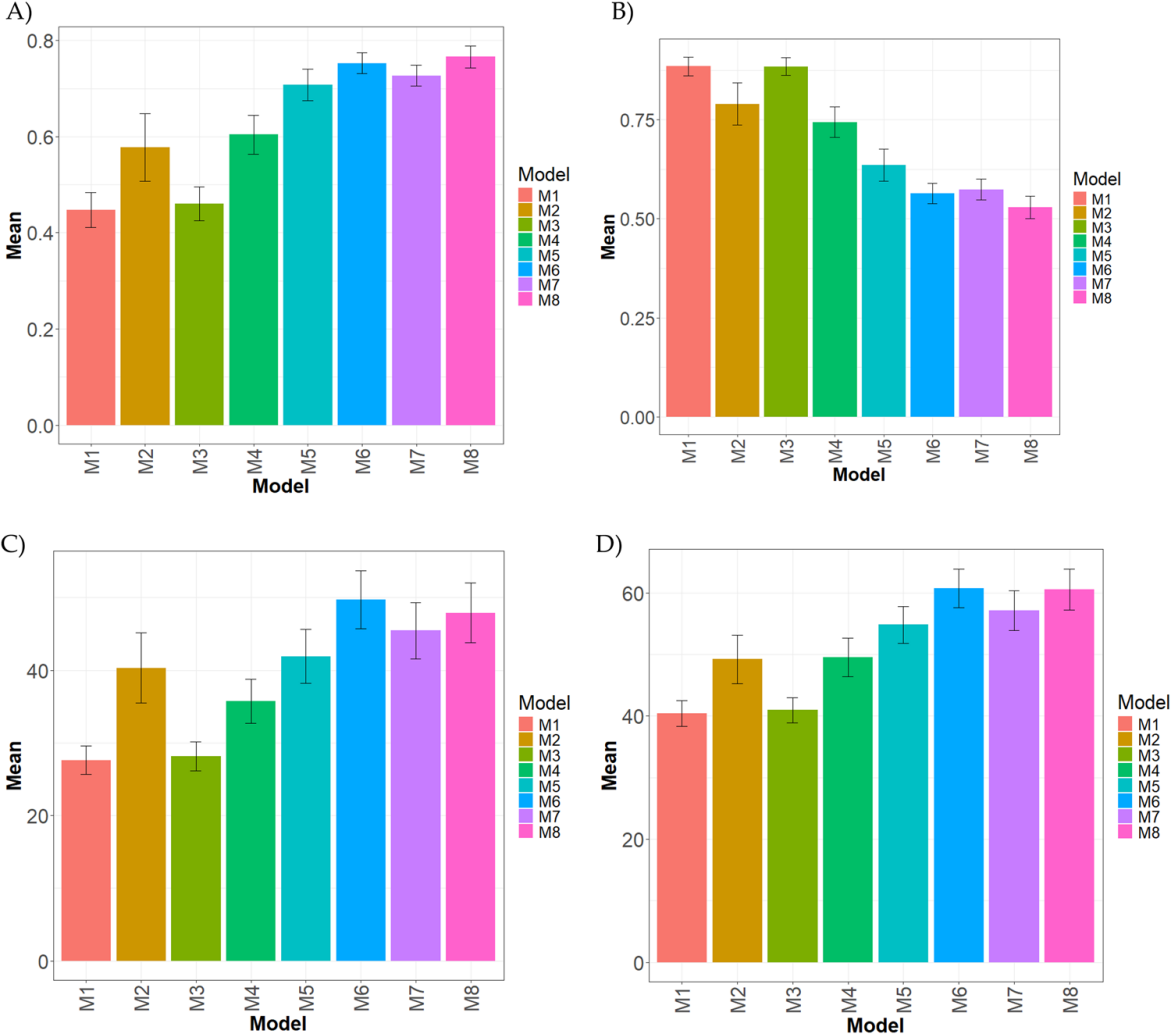
Comparative performance of genomic prediction models across environments, traits, years and type of analysis for the Wheat_WSU datasetEvaluation metrics include: (A) Pearson's correlation (COR), (B) normalized root mean square error (NRMSE), (C) percentage of matching in the top 10% (PM_10), and (D) percentage of matching in the top 20% (PM_20). Results highlight the models’ predictive performance under genotype × environment interactions. Higher COR and PM values, and lower NRMSE, indicate better generalization across environments. Model M8 consistently demonstrated superior performance across most metrics.

In Figure 3A, M8 obtained the highest correlation (0.766), with M6 (0.752, −1.85%) and M7 (0.727, −5.39%) trailing only slightly behind. M5 (0.708) showed a moderate decline (−8.19%). By contrast, M2 (0.578, −32.63%) and M4 (0.604, −26.83%) performed considerably worse, whereas M3 (0.460, −66.43%) and M1 (0.448, −71.00%) exhibited severe underperformance.

In Figure 3B, M8 achieved the lowest NRMSE (0.529), closely followed by M6 (0.564, −6.65%) and M7 (0.574, −8.55%). M5 (0.635) experienced a moderate reduction in performance (−20.08%). In contrast, M2 (0.790, −49.39%) and M4 (0.744, −40.63%) performed notably worse, whereas M3 (0.884, −67.21%) and M1 (0.885, −67.29%) showed extremely poor performance.

In Figure 3C, M6 recorded the highest PM_10 (49.748), followed closely by M8 (47.921, −3.81%) and M7 (45.452, −9.45%). M5 (41.917) experienced a moderate decrease (−18.68%). Conversely, M2 (40.326, −23.36%) and M4 (35.738, −39.20%) performed considerably worse, whereas M3 (28.150, −76.72%) and M1 (27.641, −79.98%) exhibited markedly poor results.

In Figure 3D, M6 achieved the best PM_20 (60.741), with M8 (60.578, −0.269%) and M7 (57.163, −6.26%) performing very closely. M5 (54.815) showed a moderate decline (−10.81%). By comparison, M2 (49.223, −23.40%) and M4 (49.526, −22.64%) performed substantially worse, whereas M3 (40.960, −48.29%) and M1 (40.412, −50.30%) showed the weakest performance overall.

## 4 DISCUSSION

The practical implementation of GS in plant breeding programs continues to face significant challenges. One of the foremost obstacles is the limited predictive ability of genomic models, especially under complex genetic architectures and across diverse environments (Crossa et al., 2017). Additionally, the high costs associated with genotyping and the difficulty of capturing dynamic environmental interactions constrain the broader application of GS, particularly in public breeding programs with limited resources (Rutkoski, 2019). These challenges highlight the urgent need for strategies that can improve the efficiency and accuracy of GS to ensure its sustainability and scalability in real‐world breeding contexts.

### 4.1 The role of phenomics and high‐throughput data

To address these limitations, various approaches have been proposed. Among the most promising are those that integrate complementary sources of high‐throughput data, such as phenomics, into the GS framework (Araus et al., 2018). Phenomics, through technologies such as UAS and multispectral imaging, offers the capacity to capture intermediate traits related to plant physiology, which may serve as proxies for complex traits like GY (Krause et al., 2019). Recent studies have explored the simultaneous modeling of genomic and phenomic information, demonstrating that such integration can leverage both genetic potential and dynamic phenotypic expression to improve predictive performance (Crain et al., 2018; Kunwar et al., 2025; O. A. Montesinos‐López et al., 2023).

### 4.2 Hybrid kernels as a flexible integration strategy

In this study, we proposed an alternative and flexible strategy for integrating genomic and phenomic data through the construction of hybrid kernels. By generating two novel kernels that combine the original genomic and phenomic similarity matrices, our approach seeks to exploit the complementary nature of both data sources. Genomic information reflects the inherited genetic potential of a genotype and provides a stable, heritable foundation for predictive modeling (Meuwissen et al., 2001). In contrast, phenomic data, typically derived from high‐throughput platforms such as UAV‐based imaging or proximal sensors, captures dynamic plant responses influenced by environmental variation, which are particularly useful for characterizing complex, quantitative traits (Araus & Cairns, 2014). When these two data types are modeled separately, valuable interactions and correlations between genotype and phenotype across time and space may remain unexploited. The proposed hybrid kernels are designed to capture previously unexplored information, thereby enhancing predictive ability. In particular, they exploit complementary aspects of the complex interactions between genomics and phenomics that are not adequately captured by conventional methods of integrating these two sources of information. Unlike the traditional Hadamard product approach, the proposed hybrid kernels are constructed through the subproduct of a matrix multiplication, which ensures the validity of the resulting kernels and provides a novel framework for modeling these interactions.

Among the eight implemented models, M1 and M3 relied exclusively on genomic information, whereas M2 was based solely on phenomic data. Models M4, M5, M6, M7, and M8 were hybrid approaches that integrated both genomic and phenomic information. Notably, only models M6 through M8 employed the matrix multiplication strategy to compute hybrid kernels, as described by Cuevas et al. (2025).

Overall, the poorest predictive performance was observed in the purely genomic models (M1 and M3), which performed at least 66.45% worse than M8 in terms of COR. In contrast, the best performance was achieved by the hybrid models that combined genomic and phenomic data with our proposed framework. Among these, M8 demonstrated the highest predictive ability, outperforming M7 and M6 in terms of COR by only 5.39% and 1.85%, respectively, but showing an average advantage of 17.52% over M4 and M5, which followed the traditional approach of combining genomics and phenomics. However, it is important to note that, overall, model M5 outperformed model M4 in terms of COR by approximately 18%, indicating that the use of a Gaussian kernel to model the multispectral information significantly enhanced prediction ability in this case. These findings are in agreement with those reported by Kunwar et al. (2025).

In terms of PM_10 and PM_20, the best‐performing model was M6, which on average outperformed models M4 and M5 by 28.95% and 16.73%, respectively. However, its superiority over models M8 and M7 was more modest, with differences of less than 9.45% for PM_10 and 6.26% for PM_20. These two metrics are particularly important in plant breeding because they directly assess a model's ability to correctly identify the most promising candidate lines. PM_10 measures the percentage of true top‐performing lines captured within the top 10% of predictions, whereas PM_20 does the same for the top 20%. In practical breeding programs, resources are usually limited, and only a small fraction of lines can be advanced for further testing or selection. Therefore, a model that achieves high PM_10 and PM_20 values ensures that the most valuable lines are prioritized, reducing the risk of discarding superior genotypes and increasing the likelihood of genetic gain. In this sense, even modest improvements in these metrics can have substantial practical implications, as they translate into more efficient selection and a higher probability of success in subsequent breeding cycles.

These findings align with previous studies showing that phenomic prediction often achieves higher accuracy than GP (Wang et al., 2025; P. Y. Wu et al., 2022; C. Wu et al., 2024) and that our proposed framework for integrating this information significantly improves the prediction ability. Furthermore, our results agree with those of Graciano et al. (2025) and McBreen et al. (2025a, 2025b), who also reported that integrating genomic and phenomic information leads to superior predictive performance.

It is also important to highlight that prediction ability under the multi‐environment setting was considerably higher than in the univariate setting. This improvement can be attributed to the fact that, in the multi‐environment framework, information from different environments is treated as replicates. As a result, the models benefit from a richer and more diverse data structure, which enhances the estimation of parameters by reducing noise and improving the signal‐to‐noise ratio. Essentially, the multi‐environment setting allows the models to exploit both within‐environment variation and across‐environment correlations, leading to more precise parameter estimates and, consequently, greater predictive accuracy.

### 4.3 Theoretical advancement of the Cuevas et al. (2025) framework

The current study extends the theoretical contribution of Cuevas et al. (2025) by demonstrating that their hybrid kernel methodology, originally designed for combining genomic and pedigree data, can be generalized to fuse fundamentally distinct data sources—namely, static genomic information and dynamic phenomic traits derived from high‐throughput UAV imaging. This generalization reveals the broader applicability of the triangular matrix product approach to diverse omics combinations and supports its role as a versatile tool for enhancing predictive modeling in plant breeding.

Beyond statistical improvement, the triangular hybrid kernels (upper and lower matrix products) may offer a biologically meaningful decomposition. The upper triangular component may capture commonalities in physiological responses across genotypes, while the lower triangular part could reflect genotype‐specific interactions with local environmental factors. This interpretation opens the door to using hybrid kernels not only as predictive tools but also as exploratory instruments for understanding genotype × phenotype × environment dynamics.

### 4.4 Performance under sparse and unbalanced testing designs

One of the key advantages of hybrid kernel models is their robustness under sparse and unbalanced data conditions, which often undermine the performance of traditional GP models. By leveraging both genomic similarity and high‐resolution phenotypic expression, the hybrid approach can effectively borrow strength across genotypes and environments, thus compensating for the limited overlap in training data across years or sites. This robustness makes it particularly suitable for real‐world breeding applications.

Importantly, the hybrid kernel approach is inherently modular and extensible. While this study focused on combining genomic and phenomic kernels, the same matrix product methodology could be adapted to integrate additional layers of information, such as environmental covariates, soil properties, or even outputs from crop growth models. This opens new opportunities for the design of next‐generation prediction frameworks that more comprehensively capture the determinants of complex trait expression.

### 4.5 Computational feasibility and practical utility

Another important aspect of the proposed hybrid kernel method is its computational efficiency and interpretability. Unlike black‐box models such as deep neural networks, the hybrid kernel approach maintains transparency while delivering substantial gains in predictive ability. This balance between complexity and interpretability enhances its applicability in breeding programs where both accuracy and biological insight are required.

Our implementation of this method using data from the winter wheat breeding program at WSU yielded encouraging results. Models incorporating hybrid kernels consistently outperformed those using either genomic or phenomic kernels alone, across multiple traits and validation scenarios. These improvements in predictive ability suggest that the hybrid kernels successfully captured useful and nonredundant information from both sources, thereby increasing the overall modeling capacity. Notably, this performance gain was evident even in the presence of challenges commonly faced in practical breeding, such as data imbalance, sparse replication across environments, and limited shared genotypes among environments—conditions that often reduce prediction performance in traditional GS pipelines (Crossa et al., 2017; O. A. Montesinos‐López et al., 2022).

### 4.6 Broader relevance for genomic selection

Given the ongoing challenges associated with the practical implementation of GS—including limitations in data quality, trait complexity, and G × E modeling—integrative strategies such as the one proposed here are increasingly necessary (Spindel & McCouch, 2016). Hybrid kernel models provide a pragmatic and computationally tractable means to improve predictive ability while leveraging already available genomic and phenomic resources. Our results provide empirical evidence supporting this integration as a viable and effective approach, reinforcing the value of hybrid modeling frameworks in accelerating genetic gain and improving selection decisions in plant breeding.

### 4.7 Comparison of Hadamard and Gaussian interaction kernels

Traditional Hadamard products have been widely used to model genotype‐by‐environment interaction because of their simplicity and low computational cost. However, they assume that interaction effects are strictly linear and independent across dimensions of genetic and environmental relatedness. In contrast, Gaussian kernels leverage nonlinear similarity across both genomic and phenomic dimensions, enabling smoother and more flexible modeling of complex interactions (A. Montesinos‐López et al., 2021; Shawe‐Taylor & Cristianini, 2004). In our hybrid kernels, the environmental interaction structure is captured implicitly through the Gaussian transformation, and thus extends the Hadamard product as a special case. As expected, predictive performance of models based on Gaussian hybrid kernels (M7–M8) exceeded or matched those relying solely on element‐wise interaction products (M4–M6), indicating that Gaussian kernel‐based interaction modeling offers a more biologically realistic representation of phenotypic plasticity without requiring additional computation. See Table 3 below explicitly detailing how Hadamard‐based interaction modeling relates to the Gaussian hybrid kernel in terms of biological assumptions and predictive benefits.

**TABLE 3:** Comparison between Hadamard product and matrix multiplication for construction of hybrid kernels.

Kernel type	Modeling principle	Advantages	Limitations	Observed predictive performance in this study
**Hadamard (element‐wise) interaction kernel**	Linear combination of G × P or G × E relationships; assumes independence across dimensions	Simple, low computational cost, historically widely used in G × E genomic prediction models	Cannot capture nonlinear similarities; interaction strictly proportional to main effects	Baseline performance; improvements limited for highly complex traits (e.g., GY)
**Gaussian hybrid interaction kernel**	Nonlinear transformation of genomic and phenomic similarity enabling smooth covariance structure	Better modeling of epistasis, trait‐dependent plasticity, and continuous similarity; generalizes the Hadamard case	Slightly higher computational cost; requires bandwidth tuning	Showed equal or superior predictive ability, particularly in complex traits

Abbreviation: GY, grain yield.

### 4.8 Practical implications of combining phenomics and genomics

Finally, it is important to point out that in practice, combining these data sources (genomic and phenomic) can accelerate genetic gain in several ways. For example, in early‐generation selection, HTP platforms such as multispectral imaging or UAV‐based measurements can provide indirect yet precise estimates of complex traits, which, when combined with GP, enhance the accuracy of selecting superior lines before extensive field testing. Similarly, in multi‐environment trials, integrating genomic information with phenomic data helps breeders better capture genotype × environment interactions, leading to more robust predictions across diverse growing conditions. This integration is also particularly useful for traits that are expensive or difficult to measure directly, such as root architecture or physiological responses to stress. By reducing the cost and time required for phenotyping while increasing prediction accuracy, the joint use of genomics and phenomics has the potential to substantially accelerate breeding cycles and genetic gain in wheat and other crops.

Although integrating phenomic and genomic information generally increased predictive ability in our study, the value of this improvement depends on the specific trait and its contribution to genetic gain. For highly complex traits such as GY, even moderate gains in prediction accuracy must be weighed against the operational costs and delays associated with collecting phenomic data during the crop cycle. In contrast, for simpler traits with high heritability, such as PH and heading date, UAV‐based or sensor‐based measurements can replace labor‐intensive ground phenotyping, providing substantial time and cost savings while maintaining or improving prediction ability. Thus, phenomic prediction is especially advantageous when high‐throughput measurements are readily available or serve as a substitute for manual scoring, whereas purely GP remains more cost‐efficient when phenotyping logistics restrict trait evaluation. This balance between predictive gain and resource investment should guide the practical deployment of multi‐modal selection strategies in breeding pipelines. This distinction clarifies why correlations from genomic models primarily reflect additive genetic merit, while phenomic‐based predictions may capture short‐term adaptive responses and micro‐environmental variation.

## 5 CONCLUSION AND FUTURE PERSPECTIVES

Our results show empirical evidence that the hybrid kernel approach represents a promising avenue for addressing current limitations in GS. As phenomic technologies continue to mature and become more widely adopted, the integrative modeling of genomics and phenomics is likely to become a cornerstone of next‐generation plant breeding programs. These findings are particularly encouraging for the broader and more effective implementation of GS in breeding programs. The ability of hybrid kernels to enhance model performance suggests that complementary information between genomic and phenomic data can be effectively harnessed using kernel‐based learning methods. Furthermore, this approach does not require complex or computationally intensive model modifications, making it accessible and practical for use in breeding pipelines.

In conclusion, the results of this study support the integration of genomic and phenomic data using hybrid kernels as a viable and effective strategy to enhance the predictive power of GS models. As HTP becomes increasingly available and affordable, methods like the one proposed here may play a critical role in advancing the efficiency and impact of GS in plant breeding.

## AUTHOR CONTRIBUTIONS

**Osval A. Montesinos‐López**: Conceptualization; formal analysis; investigation; methodology; writing—original draft; writing—review and editing. **Joel A. Martínez‐Regalado**: Software; validation; writing—review and editing. **Cintia Leonora Murillo‐Avalos**: Methodology; software; writing—review and editing. **Andrew W. Herr**: Visualization; writing—original draft; writing—review and editing. **Abelardo Montesinos‐López**: Conceptualization; formal analysis; investigation; writing—original draft; writing—review and editing. **Iván Delgado‐Enciso**: Funding acquisition; investigation; validation; visualization; writing—review and editing. **Jaime Cuevas**: Visualization; writing—review and editing. **José Crossa**: Conceptualization; writing—original draft; writing—review and editing. **Arron H. Carter**: Conceptualization; data curation; funding acquisition; project administration; resources; software; supervision; writing—original draft; writing—review and editing.

## CONFLICT OF INTEREST STATEMENT

The authors declare no conflicts of interest.

## APPENDIX A

### A.1

**TABLE A1:** Comparative performance of genomic prediction models in terms of Pearson's correlation (COR) (A), normalized root mean square error (NRMSE) (B), and percentage of matching in top 10% (PM_10) (C) and top 20% (PM_20) (D) for the uni‐environment results.

Year	Model	Metric	Minimum	Mean	Median	Maximum	SE	RE
2019	M1	COR	0.179	0.415	0.484	0.554	0.0425	60.72
2019	M2	COR	0.205	0.549	0.431	0.959	0.0913	21.49
2019	M3	COR	0.183	0.426	0.499	0.554	0.0435	56.57
2019	M4	COR	0.323	0.63	0.559	0.961	0.0738	5.87
2019	M5	COR	0.315	0.658	0.561	0.991	0.0708	1.37
2019	M6	COR	0.386	0.66	0.554	0.978	0.0675	1.06
2019	M7	COR	0.327	0.667	0.58	0.98	0.0676	0.00
2019	M8	COR	0.334	0.667	0.579	0.992	0.0691	0.00
2019	M1	NRMSE	0.843	0.906	0.88	0.999	0.0178	39.17
2019	M2	NRMSE	0.287	0.757	0.963	1.005	0.0914	16.28
2019	M3	NRMSE	0.847	0.901	0.877	0.992	0.0171	38.40
2019	M4	NRMSE	0.279	0.697	0.846	0.956	0.0826	7.07
2019	M5	NRMSE	0.133	0.658	0.834	0.956	0.0924	1.08
2019	M6	NRMSE	0.209	0.671	0.848	0.932	0.0859	3.07
2019	M7	NRMSE	0.2	0.663	0.823	0.954	0.0856	1.84
2019	M8	NRMSE	0.127	0.651	0.822	0.951	0.0920	0.00
2019	M1	PM_10	17.192	25.353	22.564	44.726	2.6375	48.28
2019	M2	PM_10	1.233	22.877	19.231	55.343	5.5000	64.33
2019	M3	PM_10	17.821	26.045	21.282	45.274	2.8952	44.34
2019	M4	PM_10	18.947	33.707	30.548	66.301	4.2853	11.53
2019	M5	PM_10	22.5	35.343	33.562	66.507	3.7201	6.37
2019	M6	PM_10	24.737	37.546	34.103	65.822	3.7670	0.13
2019	M7	PM_10	25.921	36.476	32.949	64.589	3.5618	3.06
2019	M8	PM_10	25.769	37.593	36.164	66.027	3.5877	0.00
2019	M1	PM_20	24.897	36.482	38.219	51.918	2.4515	37.70
2019	M2	PM_20	15.171	33.019	25.705	78.39	5.7132	52.15
2019	M3	PM_20	28.562	37.528	40.128	53.253	2.4360	33.87
2019	M4	PM_20	32	44.939	41.267	78.664	3.8532	11.79
2019	M5	PM_20	38.141	47.971	45.128	81.233	3.7539	4.72
2019	M6	PM_20	39.68	49.425	45.4	81.438	3.7972	1.64
2019	M7	PM_20	36.859	49.039	47.133	81.336	3.7896	2.44
2019	M8	PM_20	37.756	50.237	47.2	82.637	3.9052	0.00
2020	M1	COR	−0.106	0.22	0.183	0.632	0.0683	157.27
2020	M2	COR	−0.08	0.566	0.657	0.992	0.1105	0.00
2020	M3	COR	−0.146	0.235	0.219	0.636	0.0682	140.85
2020	M4	COR	−0.143	0.344	0.367	0.946	0.0891	64.53
2020	M5	COR	−0.156	0.489	0.524	0.971	0.0924	15.75
2020	M6	COR	−0.12	0.443	0.441	0.911	0.0963	27.77
2020	M7	COR	−0.166	0.368	0.339	0.895	0.0949	53.80
2020	M8	COR	−0.176	0.493	0.516	0.973	0.0975	14.81
2020	M1	NRMSE	0.787	0.998	1.026	1.115	0.0317	19.38
2020	M2	NRMSE	0.129	0.915	1.003	1.083	0.0666	9.45
2020	M3	NRMSE	0.799	0.991	1.02	1.109	0.0294	18.54
2020	M4	NRMSE	0.312	0.932	0.978	1.093	0.0579	11.48
2020	M5	NRMSE	0.24	0.851	0.892	1.149	0.0627	1.79
2020	M6	NRMSE	0.417	0.89	0.945	1.083	0.0623	6.46
2020	M7	NRMSE	0.452	0.909	0.98	1.103	0.0614	8.73
2020	M8	NRMSE	0.234	0.836	0.88	1.143	0.0687	0.00
2020	M1	PM_10	1.667	19.565	20.481	50.27	3.1479	112.04
2020	M2	PM_10	2.885	41.485	44.762	85	7.7352	0.00
2020	M3	PM_10	8.333	19.596	18.452	50.405	3.0469	111.70
2020	M4	PM_10	6.667	26.982	20.357	60.27	4.8606	53.75
2020	M5	PM_10	6.667	30.612	30	64.73	4.7147	35.52
2020	M6	PM_10	6.667	33.417	26.081	69.46	5.9131	24.14
2020	M7	PM_10	8.333	26.939	20.541	62.568	4.6797	54.00
2020	M8	PM_10	5	30.284	30	64.46	4.8048	36.99
2020	M1	PM_20	9	30.952	31.072	60.135	3.1028	64.82
2020	M2	PM_20	13	51.015	54.857	85	7.2302	0.00
2020	M3	PM_20	14	31.843	30.5	59.932	3.0178	60.21
2020	M4	PM_20	10	37.281	29.464	67.973	5.0121	36.84
2020	M5	PM_20	15	42.959	41.414	71.786	5.1058	18.75
2020	M6	PM_20	13	43.127	39.298	82.095	6.0260	18.29
2020	M7	PM_20	10	38.807	34.462	81.014	5.3913	31.46
2020	M8	PM_20	10	43.391	42	81.419	5.6711	17.57
2021	M1	COR	0.145	0.348	0.316	0.827	0.0552	76.15
2021	M2	COR	−0.066	0.532	0.724	0.986	0.1284	15.23
2021	M3	COR	0.139	0.348	0.302	0.834	0.0568	76.15
2021	M4	COR	0.119	0.464	0.42	0.878	0.0646	32.11
2021	M5	COR	−0.07	0.589	0.727	0.907	0.0876	4.07
2021	M6	COR	0.186	0.597	0.638	0.948	0.0744	2.68
2021	M7	COR	0.178	0.554	0.59	0.942	0.0684	10.65
2021	M8	COR	0.044	0.613	0.739	0.934	0.0880	0.00
2021	M1	NRMSE	0.608	0.967	1.001	1.108	0.0367	27.57
2021	M2	NRMSE	0.262	0.897	1.003	1.127	0.0664	18.34
2021	M3	NRMSE	0.628	0.966	0.996	1.085	0.0346	27.44
2021	M4	NRMSE	0.389	0.91	0.983	1.09	0.0536	20.05
2021	M5	NRMSE	0.434	0.784	0.741	1.153	0.0615	3.43
2021	M6	NRMSE	0.36	0.84	0.927	1.092	0.0636	10.82
2021	M7	NRMSE	0.39	0.844	0.935	1.106	0.0600	11.35
2021	M8	NRMSE	0.401	0.758	0.759	1.141	0.0678	0.00
2021	M1	PM_10	10	20.135	18.229	49.286	2.6309	119.10
2021	M2	PM_10	0.5	44.115	41.595	90	8.9309	0.00
2021	M3	PM_10	10	20.438	16.583	52.143	3.1236	115.85
2021	M4	PM_10	8.333	26.936	24	65	4.4183	63.78
2021	M5	PM_10	8.333	32.141	27.738	71.5	4.8715	37.25
2021	M6	PM_10	11.667	35.912	32.393	64	5.2153	22.84
2021	M7	PM_10	13.125	27.884	25.357	50.5	3.1606	58.21
2021	M8	PM_10	10.938	32.667	29.167	69	4.7001	35.04
2021	M1	PM_20	18	33.093	32.371	53.571	2.5229	58.95
2021	M2	PM_20	9.516	52.6	52.857	88.75	8.1228	0.00
2021	M3	PM_20	20	32.769	31.403	53.929	2.6128	60.52
2021	M4	PM_20	16	40.513	36.362	79.25	4.5688	29.83
2021	M5	PM_20	18	49.706	46.776	86.75	5.5782	5.82
2021	M6	PM_20	17	50.556	54.553	77.75	4.9148	4.04
2021	M7	PM_20	18	43.687	46	66	3.6634	20.40
2021	M8	PM_20	20	51.205	48.464	87	5.4902	2.72

*Note*: The last column shows the relative efficiency (RE) in percentage for each metric and year of the best model regarding the other models.

**TABLE A2:** Comparative performance of genomic prediction models in terms of Pearson's correlation (COR) (A), normalized root mean square error (NRMSE) (B), and percentage of matching in top 10% (PM_10) (C) and top 20% (PM_20) (D) for multi‐environment results.

Year	Model	Method	Metric	Minimum	Mean	Median	Maximum	SE	RE
2019	M1	G	COR	0.306	0.346	0.337	0.396	0.026	174.63
2019	M2	H	COR	0.848	0.870	0.881	0.882	0.011	9.24
2019	M3	K	COR	0.314	0.368	0.388	0.402	0.027	158.36
2019	M4	KH	COR	0.348	0.715	0.898	0.898	0.184	33.03
2019	M5	KK_H	COR	0.880	0.921	0.903	0.980	0.030	3.25
2019	M6	KHCP	COR	0.930	0.949	0.952	0.965	0.010	0.16
2019	M7	KHCPH	COR	0.920	0.941	0.946	0.957	0.011	1.00
2019	M8	KK_H_CPH	COR	0.926	0.951	0.947	0.979	0.016	0.00
2019	M1	G	NRMSE	0.937	0.964	0.969	0.987	0.015	219.02
2019	M2	H	NRMSE	0.478	0.563	0.510	0.700	0.070	86.11
2019	M3	K	NRMSE	0.928	0.952	0.951	0.978	0.014	214.95
2019	M4	KH	NRMSE	0.450	0.626	0.481	0.948	0.161	107.15
2019	M5	KK_H	NRMSE	0.197	0.380	0.436	0.508	0.094	25.80
2019	M6	KHCP	NRMSE	0.265	0.315	0.307	0.374	0.032	4.27
2019	M7	KHCPH	NRMSE	0.296	0.340	0.326	0.399	0.030	12.50
2019	M8	KK_H_CPH	NRMSE	0.200	0.302	0.322	0.385	0.054	0.00
2019	M1	G	PM_10	22.009	30.819	33.929	36.518	4.468	99.95
2019	M2	H	PM_10	5.357	40.045	18.527	96.250	28.359	53.88
2019	M3	K	PM_10	22.902	32.515	34.911	39.732	5.004	89.52
2019	M4	KH	PM_10	23.304	31.816	35.580	36.563	4.265	93.68
2019	M5	KK_H	PM_10	27.277	53.824	38.125	96.071	21.354	14.49
2019	M6	KHCP	PM_10	40.625	61.622	48.259	95.982	17.321	0.00
2019	M7	KHCPH	PM_10	39.196	60.164	45.625	95.670	17.850	2.42
2019	M8	KK_H_CPH	PM_10	40.179	60.610	45.268	96.384	17.947	1.67
2019	M1	G	PM_20	29.844	38.505	36.518	49.152	5.662	60.65
2019	M2	H	PM_20	36.094	43.341	39.576	54.353	5.597	42.73
2019	M3	K	PM_20	32.455	39.836	37.121	49.933	5.225	55.29
2019	M4	KH	PM_20	35.156	44.933	45.134	54.509	5.588	37.67
2019	M5	KK_H	PM_20	45.781	55.543	58.036	62.813	5.072	11.37
2019	M6	KHCP	PM_20	60.201	60.446	60.290	60.848	0.203	2.34
2019	M7	KHCPH	PM_20	57.411	59.427	59.844	61.027	1.065	4.09
2019	M8	KK_H_CPH	PM_20	57.522	61.860	60.871	67.188	2.834	0.00
2020	M1	G	COR	0.475	0.659	0.736	0.767	0.093	42.20
2020	M2	H	COR	0.296	0.737	0.938	0.978	0.221	27.08
2020	M3	K	COR	0.511	0.672	0.733	0.772	0.082	39.49
2020	M4	KH	COR	0.507	0.787	0.915	0.939	0.140	19.07
2020	M5	KK_H	COR	0.594	0.841	0.959	0.970	0.123	11.43
2020	M6	KHCP	COR	0.856	0.931	0.966	0.972	0.038	0.63
2020	M7	KHCPH	COR	0.841	0.928	0.962	0.980	0.044	1.00
2020	M8	KK_H_CPH	COR	0.857	0.937	0.967	0.987	0.040	0.00
2020	M1	G	NRMSE	0.669	0.751	0.688	0.896	0.073	141.58
2020	M2	H	NRMSE	0.211	0.518	0.350	0.992	0.241	66.46
2020	M3	K	NRMSE	0.692	0.769	0.713	0.902	0.067	147.17
2020	M4	KH	NRMSE	0.349	0.537	0.374	0.888	0.176	72.64
2020	M5	KK_H	NRMSE	0.198	0.437	0.288	0.824	0.195	40.35
2020	M6	KHCP	NRMSE	0.224	0.338	0.261	0.529	0.096	8.68
2020	M7	KHCPH	NRMSE	0.193	0.339	0.274	0.550	0.108	8.94
2020	M8	KK_H_CPH	NRMSE	0.152	0.311	0.257	0.524	0.111	0.00
2020	M1	G	PM_10	25.164	35.164	34.508	45.820	5.972	80.11
2020	M2	H	PM_10	30.246	45.082	46.066	58.934	8.296	40.48
2020	M3	K	PM_10	28.853	36.913	37.541	44.344	4.483	71.57
2020	M4	KH	PM_10	36.066	48.934	51.475	59.262	6.816	29.43
2020	M5	KK_H	PM_10	44.508	51.585	52.623	57.623	3.821	22.77
2020	M6	KHCP	PM_10	51.967	62.842	64.672	71.885	5.822	0.78
2020	M7	KHCPH	PM_10	51.475	61.202	62.459	69.672	5.290	3.48
2020	M8	KK_H_CPH	PM_10	56.885	63.333	64.590	68.525	3.418	0.00
2020	M1	G	PM_20	46.312	54.727	47.951	69.918	7.610	48.30
2020	M2	H	PM_20	47.213	69.549	64.426	97.008	14.601	16.70
2020	M3	K	PM_20	48.361	55.943	48.975	70.492	7.277	45.08
2020	M4	KH	PM_20	62.459	73.429	63.566	94.262	10.422	10.53
2020	M5	KK_H	PM_20	56.271	74.071	68.320	97.623	12.279	9.57
2020	M6	KHCP	PM_20	71.353	81.161	75.000	97.131	8.054	0.00
2020	M7	KHCPH	PM_20	66.967	78.456	71.025	97.377	9.533	3.45
2020	M8	KK_H_CPH	PM_20	69.303	80.041	72.213	98.607	9.321	1.40
2021	M1	G	COR	0.654	0.739	0.750	0.814	0.046	31.48
2021	M2	H	COR	−0.095	0.218	0.148	0.601	0.204	346.58
2021	M3	K	COR	0.676	0.756	0.763	0.829	0.045	28.63
2021	M4	KH	COR	0.726	0.769	0.740	0.842	0.037	26.37
2021	M5	KK_H	COR	0.739	0.789	0.775	0.851	0.033	23.30
2021	M6	KHCP	COR	0.962	0.972	0.970	0.984	0.006	0.00
2021	M7	KHCPH	COR	0.954	0.968	0.968	0.982	0.008	0.41
2021	M8	KK_H_CPH	COR	0.956	0.969	0.968	0.982	0.008	0.37
2021	M1	G	NRMSE	0.598	0.691	0.692	0.783	0.053	177.96
2021	M2	H	NRMSE	0.919	1.024	1.064	1.090	0.053	311.95
2021	M3	K	NRMSE	0.595	0.696	0.702	0.791	0.057	179.97
2021	M4	KH	NRMSE	0.584	0.659	0.683	0.711	0.039	165.25
2021	M5	KK_H	NRMSE	0.569	0.638	0.645	0.699	0.038	156.44
2021	M6	KHCP	NRMSE	0.195	0.249	0.259	0.292	0.028	0.00
2021	M7	KHCPH	NRMSE	0.205	0.262	0.262	0.318	0.033	5.27
2021	M8	KK_H_CPH	NRMSE	0.206	0.260	0.263	0.312	0.031	4.59
2021	M1	G	PM_10	17.703	37.297	39.324	54.865	10.776	82.49
2021	M2	H	PM_10	20.946	38.514	46.757	47.838	8.789	76.72
2021	M3	K	PM_10	14.730	36.081	38.514	55.000	11.689	88.64
2021	M4	KH	PM_10	17.297	49.234	58.243	72.162	16.466	38.24
2021	M5	KK_H	PM_10	18.784	49.595	56.216	73.784	16.219	37.24
2021	M6	KHCP	PM_10	24.054	68.063	88.649	91.487	22.020	0.00
2021	M7	KHCPH	PM_10	16.216	64.144	87.703	88.514	23.965	6.11
2021	M8	KK_H_CPH	PM_10	15.946	65.496	88.784	91.757	24.790	3.92
2021	M1	G	PM_20	35.000	50.434	56.233	60.069	7.796	57.90
2021	M2	H	PM_20	34.384	36.690	37.329	38.356	1.191	117.05
2021	M3	K	PM_20	34.726	50.137	55.069	60.616	7.870	58.83
2021	M4	KH	PM_20	37.466	58.333	68.630	68.904	10.434	36.52
2021	M5	KK_H	PM_20	40.548	58.219	66.644	67.466	8.839	36.79
2021	M6	KHCP	PM_20	50.274	79.635	92.671	95.959	14.711	0.00
2021	M7	KHCPH	PM_20	41.164	76.370	92.055	95.890	17.638	4.28
2021	M8	KK_H_CPH	PM_20	42.123	76.735	92.055	96.027	17.344	3.78

*Note*: The last column shows the relative efficiency (RE) in percentage for each metric and year of the best model regarding the other models. Please see Table 2 for the descriptions of genomic prediction models and their kernel configurations.
